# All‐Biomass Nanocomposite Films via Facile and Sustainable Design Procedure for Thermal Management and Electromagnetic Interference Shielding

**DOI:** 10.1002/advs.202510372

**Published:** 2025-08-27

**Authors:** Junchao Ren, Chenglei Huang, Rui Tan, Jianlong Chen, Mengde Huang, Mingfeng Wang, Weiwei Liu, Bin Li, Zhong Ma, Lu Wang, Hanwu Lei, Erguang Huo, Qingfa Zhang

**Affiliations:** ^1^ School of Engineering Anhui Agricultural University Hefei Anhui 230036 China; ^2^ School of Food and Biological Engineering Hefei University of Technology Hefei Anhui 230009 China; ^3^ Department of Biological Systems Engineering Washington State University Richland Washington 99354 USA; ^4^ Key Laboratory of Efficient Low‐carbon Energy Conversion and Utilization of Jiangsu Provincial Higher Education Institutions, School of Physical Science and Technology Suzhou University of Science and Technology Suzhou Jiangsu 215009 China

**Keywords:** all‐biomass, nanocomposite film, sustainability, thermal management, EMI shielding

## Abstract

Emphasizing sustainability and low‐cost flexible films to overcome thermal accumulation and electromagnetic radiation pollution is an urgent need in response to the lightweight and integrated development of electronic devices. Herein, a fully biomass‐derived nanocomposite film is proposed and fabricated by self‐assembling nano carbon spheres (NCSs) and cellulose nanofibers (CNFs) through a simple and scalable vacuum‐assisted filtration method. The results reveal that the development of ordered and graphitized structures of NCSs through high‐temperature pyrolysis facilitates *π–π* stacking, interfacial adhesion, and the construction ofconductive networks in the NCSs/CNFs films (NCF). Among all the NCF samples, NCF 5:5 achieves a tensile strength of 41.74 MPa, in‐plane thermal conductivity of 2.21 W·m^−1^·K^−1^, and EMI shielding of 23.77 dB, representing the most balanced integration of mechanical, thermal, and shielding properties. Simultaneously, it maintains infrared stealth and stable performance over 100 deformation cycles. The synergy of all‐biomass composition, green fabrication, and superior multifunctional properties highlights the potential of the NCF in flexible electronics, wearable devices, and eco‐friendly EMI shielding applications.

## Graphical abstract

All‐biomass‐derived composite films are fabricated from wheat straw–derived nanocarbon spheres and cellulose nanofibers through a simple, scalable process, which exhibit superior integrated electrical–thermal–mechanical properties and can be applied as sustainable materials for flexible electronics and green packaging.

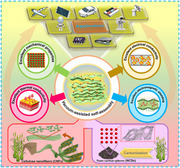

## 1 Introduction

With the arrival of the 5G information technology era and the continuous increase in the integration of electronic devices, the demand for both efficient thermal management and electromagnetic interference (EMI) shielding is steadily increasing.^[^ ^1^ ^]^ Improving thermal conductivity and EMI shielding properties has become a key technical challenge in the design of electronic devices, particularly in high‐performance computing, electric vehicles, and high‐frequency, high‐power electronic components.^[^ ^2^ ^]^ Although traditional electric heating materials, such as metal materials, ceramic materials, graphite materials, etc., exhibit excellent electrical and thermal conductivity, high density, weight, and production costs fail to allow their use in modern lightweight and low‐cost high‐performance electronics.^[^ ^3^, ^4^ ^]^ In comparison, the electrothermal polymer composites, which incorporate electric heating fillers into the polymer matrix, have attracted increasing attention due to their lightweight, flexibility, machinability, and chemical resistance.^[^ ^5^ ^]^ The previous works proved that the electrical and thermal performance can be improved by carbon nanotubes (CNT),^[^ ^6^ ^]^ graphene,^[^ ^7^ ^]^ and MXene^[^ ^8^ ^]^ as functional fillers, and the final performance is still highly dependent on film thickness and filler concentration due to the huge interface incompatibility.^[^ ^9^ ^]^ Therefore, developing novel, lightweight, low‐cost materials with superior thermal conductivity and EMI shielding properties has become the focus of research in advanced electronics and energy storage systems.^[^ ^10^ ^]^

Nano carbon spheres (NCSs) prepared from biomass precursor systems with low cost, environmental friendliness, and renewable characteristics are an ideal choice for replacing traditional materials among many new materials.^[^ ^11^ ^]^ As a renewable and sustainable material, NCSs can be readily synthesized from a wide range of sources, including wood,^[^ ^12^ ^]^ crop waste,^[^ ^13^ ^]^ and other natural organic matter.^[^ ^14^ ^]^ A highly ordered sp^2^‐hybridized carbon lattice structure and a 3D interconnected conductive network are formed in NCSs through a high‐temperature carbonization process. The multistage pore structure of NCSs, characterized by micropore–mesopore coordination, significantly increases the specific surface area, while a continuous electron transport channel is established via *π–π* electron cloud overlap.^[^ ^15^, ^16^ ^]^ In particular, NCSs are widely used in batteries, capacitors, sensors, conductive films, and other applications in high‐frequency and high‐power electronic devices due to their efficient electrical conductivity and ability to reduce power loss.^[^ ^17^ ^]^ It is worth noting that excessive heat generated inside the device as the current density increases cannot escape efficiently, which affects the performance and stability of high‐power electronic devices.^[^ ^18^ ^]^ The thermal conductivity (κ > 400 W m·K^−1^) of NCSs can efficiently conduct thermal due to the strong phonon coupling effect of its intrinsic carbon‐carbon covalent bond, improving the thermal dissipation efficiency of electronic devices.^[^ ^19^ ^]^ Although NCSs exhibit excellent thermal conductivity and EMI shielding, their relatively weak mechanical properties are prone to deformation or damage due to brittleness and high specific surface area, especially under high‐stress conditions.^[^ ^20^ ^]^ Currently, in order to improve the mechanical properties of NCSs and expand their application field, researchers have begun to combine NCSs with polymer materials to construct polymer‐flexible composite materials.^[^ ^21^ ^]^

Cellulose, as one of the most abundant natural polymer materials on earth, is primarily derived from plant resources such as wood, cotton, hemp, and straw.^[^ ^22^ ^]^ The chemical structure of cellulose consists of β‐D‐glucose units linked by β‐1,4‐glycosidic bonds, and the molecular chains are rich in hydroxyl groups, which form crystalline and amorphous regions through hydrogen bonding and intermolecular interactions.^[^ ^23^ ^]^ Benefiting from its excellent mechanical properties, low density, environmental friendliness, biodegradability, and renewability, cellulose has become an ideal substitute for synthetic materials such as polyvinyl alcohol, polyurethane foam, and steel.^[^ ^24^ ^]^ In recent years, cellulose‐based composites have demonstrated significant potential in applications such as thermal management, electronic sensors, and EMI shielding, achieved through the incorporation of conductive fillers like carbon black, metal particles, and MXene.^[^ ^25^ ^]^ However, the practical application of cellulose‐based composites still faces the defect of poor compatibility with the interface of conductive fillers, which limits the performance in applications requiring efficient conductance and thermal conductance.^[^ ^26^ ^]^ Cellulose nanofibers (CNFs), a kind of nanomaterial with a high length‐to‐diameter ratio and a single chemical composition, provide a broad space for the preparation of functional composites due to their high hydrophilicity and high modulus.^[^ ^27^ ^]^ Meanwhile, the combination of CNFs with other functional materials (such as graphene, carbon nanotubes, etc.) can further enhance their electrical conductivity, magnetic properties, and thermal management properties.^[^ ^28^ ^]^ In addition, with advancements in production processes and the reduction of costs, CNFs are expected to become an important green material that will replace traditional materials in the future and be applied in fields such as packaging materials, coatings, electronic devices, medical devices, and others, thus promoting the progress of materials science for sustainable development.^[^ ^29^ ^]^

In summary, high‐performance multifunctional NCSs/CNFs films (NCF) are fabricated through a simple and controllable vacuum‐assisted filtration method. One of the objectives of this study is to analyze the dispersion, loading capacity, and interfacial interaction of NCSs with the matrix in NCF, and its ultimate goal is to systematically explore the mechanism of NCSs on the mechanical properties, thermal conductivity, and EMI shielding of the composites. This work is expected to provide insights for the future development and application of customized and intelligent materials by offering an in‐depth analysis of the NCF mechanisms and their mechanical, thermal, and electrical properties.

## 2 Experimental Section

### 2.1 Materials

Wheat straw powder was sourced from Anqing, Anhui Province, China. Sodium chlorite (NaClO_2_, AR), potassium hydroxide (KOH, AR), oxalic acid dihydrate (H_2_C_2_O_4_·2H_2_O, AR), glacial acetic acid (CH_3_COOH, AR), and choline chloride (C_5_H_14_ClNO, AR) were obtained from Aladdin Reagent Co., Ltd. Anionic surfactant W‐920 (liquid, ≥98% purity), nonionic dispersant W‐511 (sodium polyacrylate, 30 wt.% aqueous solution), a silane coupling agent(≥ 98% purity), and a siloxane‐modified defoamer (20 wt.% active content) were all purchased from Sinopharm Chemical Reagent Co., Ltd. High‐purity ultrapure water (resistivity ≥18.2 MΩ·cm) was used throughout the experiments.

### 2.2 Preparation of Cellulose Nanofibers (CNFs)

First, 2.84 g NaClO_2_ was added to 200 mL ultrapure water, and the pH was adjusted to 4–5 using glacial acetic acid to obtain a delignification solution. Wheat straw powder was immersed in the delignification solution and reacted at 75°C for 1 h, followed by the addition of the above delignification solution to continue the reaction. This process was repeated five times. After the reaction was completed, the material was filtered and thoroughly washed with ultrapure water until a neutral pH was achieved. Subsequently, the pretreated wheat straw powder was treated with 500 mL of 2 wt.% KOH solution at 90°C for 2 h, and cellulose was obtained by freeze‐drying the material at −60°C for 48 h after washing with ultrapure water until neutral. A deep eutectic solvent (DES) was prepared by mixing oxalic acid dihydrate and choline chloride at a molar ratio of 1:1, heating at 80°C under continuous stirring until a transparent homogeneous liquid was formed. The purified cellulose was dispersed into the DES system and reacted at 110°C for 1 h. The mixture was filtered and washed thoroughly with ultrapure water to remove residual DES components. Finally, the obtained cellulose suspension was diluted with ultrapure water to a concentration of 5 mg mL^−1^ and then subjected to ultrasonication using a 900 W ultrasonic cell disruptor for 30 min to obtain cellulose nanofibers (CNFs).

### 2.3 Preparation of Nano Carbon Spheres (NCSs)

The wheat straw powder was first subjected to pre‐carbonization treatment in a muffle furnace at 800°C for 2 h with a heating rate of 5°C min^−1^ in a nitrogen atmosphere. The pre‐carbonized wheat straw powder was then treated with a 5 wt.% hydrogen peroxide (H_2_O_2_) solution for 12 h to remove surface impurities, clean the material, and prepare it for the subsequent high‐temperature carbonization at 1600°C. The dried pre‐carbonized wheat straw powder was further carbonized in a high‐temperature tube furnace at 1600°C for 2 h with a heating rate of 10°C min^−1^ under a nitrogen atmosphere. After cooling to room temperature, the carbonized material was subjected to ball milling using a bidirectional planetary ball mill at 3500 rpm for 4 h to obtain fine carbonized wheat straw powder. To improve dispersion and achieve uniform particle size reduction, the carbonized wheat straw powder (20 wt.%) was mixed with auxiliary agents including an anionic surfactant (W‐920, 5 wt.%), a nonionic polymeric dispersant (W‐511, sodium polyacrylate, 3 wt.%), a silane coupling agent (1 wt.%), and a siloxane‐modified defoamer (0.8 wt.%), with ultrapure water added to make up the remaining weight. The resulting mixture was thoroughly homogenized and subjected to ball milling in a bidirectional planetary ball mill at 4500 rpm for 4 h to reduce particle size and improve dispersibility. After ball milling, the dispersion was subjected to ultrasonication for 30 min to further break up agglomerates and improve uniformity. The resulting suspension was then centrifuged at 5000 rpm for 20 min to remove large particles and poorly dispersed material. Finally, the supernatant containing uniformly dispersed carbonized wheat straw nanoparticles was collected and freeze‐dried at −60°C for 48 h to obtain nano carbon spheres (NCSs).

### 2.4 Preparation of Nano Carbon Spheres/Cellulose Nanofibers Composite Films

**Figure 1:**
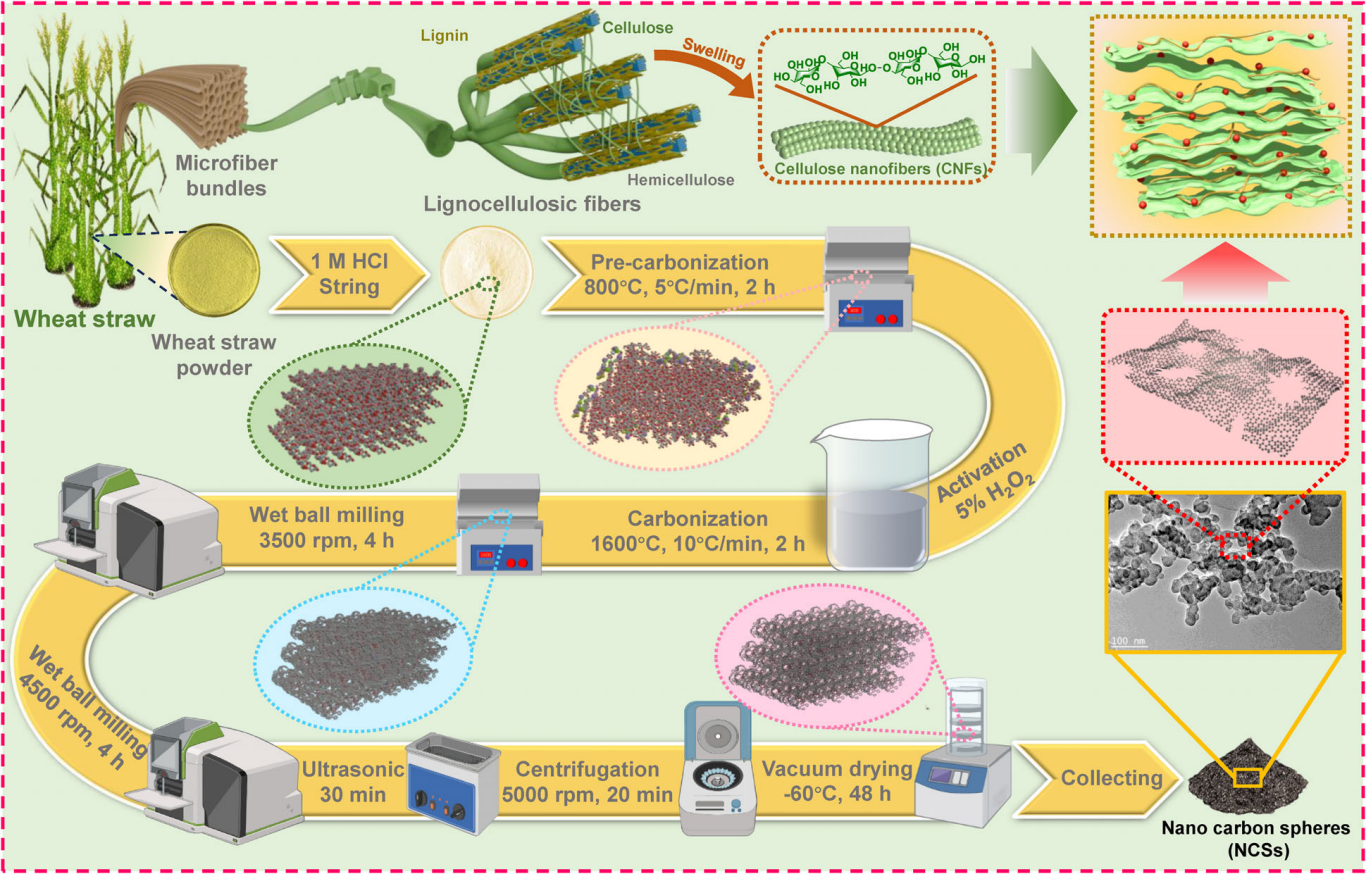
Schematic diagram of the synthesis of CNFs, NCSs, and NCSs/CNFs composite films.

NCSs were added to the CNFs suspension (5 mg mL^−1^) at various mass ratios of CNFs to NCSs (9:1, 7:3, 5:5, 3:7, and 1:9). The mixtures were magnetically stirred to obtain homogeneous suspensions, which were then subjected to vacuum filtration to form films. The resulting wet films were sandwiched between two glass plates and dried at 60°C for 24 h to obtain the NCSs/CNFs composite films. The films with different compositions were designated as NCF 9:1, NCF 7:3, NCF 5:5, NCF 3:7, NCF 1:9, respectively. For comparison, a 20 mL CNFs suspension (5 mg mL^−1^) was subjected to vacuum filtration to form cellulose nanofiber films (CF), and 100 mg of NCSs was also subjected to vacuum filtration to form NCS films (NCSF) as illustrated in **Figure** **1**.

### 2.5 Characterizations

The microstructure of NCSs, CNFs, and NCF were characterized by an SEM (Quanta 250, USA) operated at 10 kV acceleration voltage. TEM (JEM‐2100F, JEOL, Japan) was employed to study the nanostructure of CNFs and NCSs. XRD (SmartLab SE, Rigaku, Japan) measurements were performed for crystallization analysis with a scanning range of 2θ = 10°–70°. FT‐IR (Nicolet IS50, Thermo Scientific, USA) measurements were performed with a 400–4000 cm^−1^ wavenumber range to identify the functional groups. Raman spectroscopy (Horiba jobin yvon, France) was employed to evaluate the graphitization degree of the carbon‐based materials, while X‐ray photoelectron spectroscopy (XPS, K‐Alpha, Thermo Scientific, USA) was used to analyze their surface elemental composition and chemical states. The specific surface area and pore structure of the NCSs were analyzed using nitrogen adsorption–desorption isotherms measured by the Brunauer–Emmett–Teller (BET) method (Micromeritics ASAP 2460, USA). TGA measurements (TG 209 F1, NETZSCH, Germany) were carried out from room temperature to 800°C at a heating rate of 10°C min^−1^ under nitrogen flow to assess the thermal stability and residual mass of NCSs, CF, and NCF. Water contact angles were measured using a standard goniometer (Biolin Scientific, Attension Theta, Germany).

Tensile tests were performed on dumbbell‐shaped CF and NCF specimens using a tensile testing machine (UTM4104X, Sunstest Co., Ltd., China) with a rate of 5 mm min^−1^. The final tensile properties were determined by averaging the results of five individual measurements. The thermal conductivity (TC) was calculated using the equation: TC = α × ρ × Cp, where α, ρ, and Cp represent the thermal diffusivity, density, and specific heat capacity of the samples, respectively. The thermal diffusivity (α) was measured using a laser flash analyzer (LFA 447, NETZSCH, Germany), the density (ρ) was determined by weighing and measuring the volume of the samples, and the specific heat capacity (Cp) was measured by differential scanning calorimetry (Q2000, USA) using the sapphire method. The thermal dissipation performance was validated by monitoring the surface temperature of CF and NCF using an IR camera (E4, USA) during the heating and cooling processes.

The Joule heating performance of the films was tested at different voltages using a DC power supply. The electrical conductivity performance of CF and NCF was measured by a four‐point probe instrument (RTS‐8, China). The electromagnetic interference (EMI) shielding performance of CF and NCF, such as S11 and S21 of different samples, was evaluated using a vector network analyzer (Agilent N5232A, USA) over the X‐band frequency range of 8.2–12.4 GHz. Before measurement, each sample was cut to match the dimensions of the waveguide and fixed securely between two flange‐connected waveguides. The value of SET, SER, and SEA can be calculated through the following equations:^[^ ^12^ ^]^

(1) $$R={\left|S_{11}\right|}^{2}$$

(2) $$T={\left|S_{21}\right|}^{2}$$

(3) A=1−R−T

(4) $$SE_{R}=-10\times \mathrm{lg}\left(1-R\right)$$

(5) $$SE_{A}=-10\times \mathrm{lg}\left(\frac{T}{1-R}\right)$$

(6) $$SE_{T}=-10\times \mathrm{lg}\left(T\right)=SE_{R}+SE_{A}+SE_{M}$$

where R, T, and A corresponded to the EMI reflection coefficient, EMI transmission coefficient, and EMI absorption coefficient, respectively.

## 3 Results and Discussion

**Figure 2:**
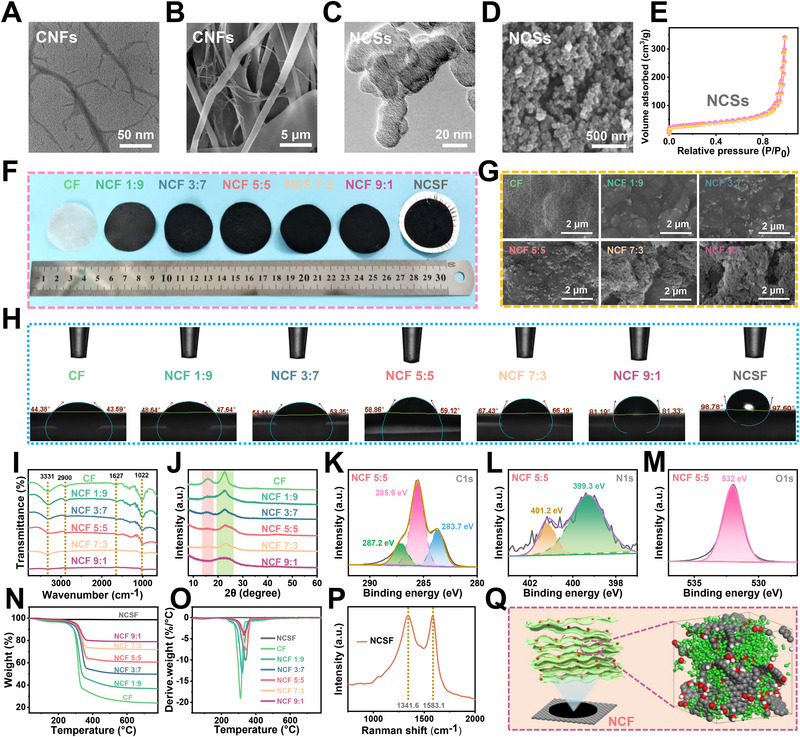
A) TEM image and B) SEM image of CNFs. C) TEM image and D) SEM image of NCSs. E) Nitrogen adsorption–desorption isotherms of NCSs. F) Digital photographs, G) SEM images, H) water contact angles, I) FTIR spectra, and J) XRD patterns of CF, NCF 1:9, NCF 3:7, NCF 5:5, NCF 7:3, and NCF 9:1. K–M) XPS spectra of C 1s, N 1s, and O 1s of NCF 5:5. N) TG and O) DTG curves of all composite films. P) Raman spectrum of NCFs. Q) Schematic illustration of the interaction mechanism between NCSs and CNFs.

As shown in the TEM image (**Figure** **2**A), the prepared cellulose nanofibers (CNFs) exhibit a rod‐like morphology with diameters of ≈5–20 nm, a high aspect ratio, smooth surfaces, and clearly defined boundaries, which demonstrates the successful disintegration of cellulose into the nanoscale during the treatment process. Also, Figure 2B shows that the network‐like structural features of the CNFs provide a robust structural basis for their further application in composite materials by establishing a continuous phase. Figure 2C shows a disordered layered morphology with partially transparent regions in the NCSs (≈20 nm in size), indicating the presence of thin graphene‐like structures. Meanwhile, the SEM image (Figure 2D) reveals a loose and fluffy texture with a spherical structure due to the fact that the combined effects of particle agglomeration caused by the high specific surface area, irregular accumulation of particles, and the curling of heat‐treated lamellar layers were achieved. The nitrogen adsorption–desorption isotherm of the NCSs shown in Figure 2E is a typical type IV isotherm with a pronounced hysteresis loop, indicating the presence of mesoporous structures. The specific surface area reaches 108.02 m^2^ g^−1^, which reflects high porosity and large surface area. This is consistent with the SEM and TEM results. Photographs of the as‐prepared CF, NCF, and NCSF are shown in Figure 2F. CF exhibited relatively uniform color and texture, indicating good film‐forming ability and homogeneous component distribution of CNFs.

As the NCSs content increased, the films became progressively darker due to the intrinsic blackness and strong light absorption of the carbon materials. However, visible particle agglomeration and increasingly nonuniform distribution appeared on the surface of the NCF 3:7 and NCF 1:9. The NCF 5:5 exhibited the most uniform color and appearance. It is worth noting that NCSF cannot form a stable self‐supporting film due to the lack of sufficient interparticle cohesion and network‐forming capability. The SEM images of the CF and NCF prepared by varying the ratio (0:10, 1:9, 3:7, 5:5, 7:3, and 9:1) are shown in Figure 2G and Figure S3 (Supporting Information). The CF is relatively smooth, and a distinct nanocellulose network can be observed. As the NCSs' content increases, slight particle aggregation is observed in NCF 1:9 and NCF 3:7, which can be attributed to the limited dispersion of low‐content NCSs within the matrix. The NCF 5:5 exhibits the most favorable dispersion with uniformly distributed carbon particles, a dense and homogeneous surface. In contrast, obvious carbon particle agglomeration and uneven surface distribution are observed in NCF 7:3 and NCF 9:1 due to the fact that particle agglomeration is caused by the poor dispersion of high‐concentration NCSs. In the case of NCF 5:5, the carbon particles are well‐embedded within the fibrous network, showing strong interfacial bonding and minimal void formation. This well‐organized and interconnected microstructure not only facilitates the formation of continuous conductive pathways for electron transport but also enhances mechanical reinforcement by enabling effective stress transfer between the components. On the other hand, NCF 9:1 shows obvious carbon particle agglomerates, large inter‐particle gaps, and poor integration with the nanocellulose matrix, which may hinder electrical and thermal conductivity and weaken mechanical performance due to stress concentration points and interfacial defects (Figure S4, Supporting Information). In addition to SEM, atomic force microscopy (AFM) was performed to provide further evidence of the surface microstructure and topography at the nanoscale. The AFM image of the NCF5:5 is presented in Figure S5 (Supporting Information). It reveals a relatively rough and compact surface with a uniform distribution of nanoscale features. The average surface roughness (Ra) of NCF5:5 was measured to be 136 nm, indicating a relatively rough and textured surface at the nanoscale. The effective contact area of conductive domains can be enhanced by the increased surface roughness, thereby promoting the formation of efficient electron transport pathways and improving the overall electrical conductivity. Moreover, phonon transport efficiency is enhanced due to the increased nanoscale roughness arising from the intimate contact between CNSs and CNFs, resulting in an improvement in thermal conductivity. Similarly, the enhanced electromagnetic shielding effectiveness is attributed to the homogeneous and roughened surface structure, which promotes electromagnetic wave scattering and absorption. Additionally, the uniform dispersion and tight interfacial bonding contribute to the observed improvement in mechanical strength and flexibility by suppressing local stress concentrations and microcrack initiation. As shown in Figure 2H, the contact angles of CF, NCF, and NCSF present a gradual increasing trend with the rise of NCSs content. This behavior can be attributed to the inherent hydrophilicity of CNFs, which contain abundant polar functional groups capable of forming hydrogen bonds with water molecules. As the NCSs content increases, the introduction of hydrophobic carbon particles reduces the exposure of these polar sites on the film surface, thereby diminishing its wettability. Figure 2I displays the FTIR spectra of the CF and NCF. As can be observed that the broad and intense characteristic peaks at 3331 and 1627 cm^−1^ are primarily attributed to the –OH stretching vibration.^[^ ^30^ ^]^ The peak at 1022 cm^−1^ because to the stretching vibration of C–O–C, which reflects the skeleton structure of CNFs. The absorption peak of 2900 cm^−1^ corresponds to the stretching vibration of –CH_2_–. Notably, the intensities of all characteristic peaks gradually decrease with increasing NCSs content due to the fact that the introduced NCSs suppress the exposure of polar functional groups on the CNFs surface.^[^ ^31^ ^]^ The XRD patterns presented in Figure 2J reveal the structural characteristics of the CF and NCF. The CF exhibits a prominent diffraction peak at ≈22.7° corresponding to the (200) plane of cellulose I, which reflects the semi‐crystalline nature of the CNFs. Also, a smaller peak is observed at ≈15.7°, which is associated with the cellulose crystalline structure.^[^ ^32^ ^]^ It should be noted that the intensity of both peaks gradually decreases with increasing NCSs content in NCF, indicating that the introduction of NCSs leads to a reduction in overall crystallinity. This observation is consistent with the FTIR results. The X‐ray photoelectron spectra shown in Figure 2K–M reveal the presence of carbon, nitrogen, and oxygen on the surface of NCF. In the C 1s spectrum, three main peaks are observed at 283.7, 285.6, and 287.2 eV corresponding to C‐C, C‐O, and C═O, respectively. In the N 1s spectrum, two peaks are observed at 399.3 and 401.2 eV, attributed to pyridine nitrogen and amine nitrogen (–NH_2_), respectively. The O 1s spectrum shows a peak at 532 eV assigned to hydroxyl (–OH), indicating the presence of oxygen functionalities on the surface. Figure 2N–O shows the TG and DTG curves of CF, NCF, and NCSF. CF and NCF present a slight weight loss at ≈150°C attributed to moisture evaporation and the initial thermal decomposition of CNFs. The maximum weight loss observed between 300 and 380°C corresponds to the thermal decomposition of cellulose. It should be noted that the residual carbon content increases and the maximum decomposition peak temperature shifts upward as the NCS_S_ content in NCF gradually increases (Figure 2O), indicating that the thermal stability of the composite films is effectively enhanced by the addition of NCS_S_. The Raman spectrum of the NCSF shown in Figure 2P exhibits two distinct peaks with the D band at 1341.6 cm^−1^ and the G band at 1583.1 cm^−1^. The intensity ratio of the D band to the G band (I_D/I_G) is 0.998, which indicates a good combination of sp^2^ hybridized carbon atoms and defect sites. The enhancement of interfacial adhesion, the facilitation of uniform dispersion, and the formation of continuous conductive networks were achieved by hydrogen bonding and *π–π* stacking interactions between NCSs and CNFs, as shown in Figure 2Q.

**Figure 3:**
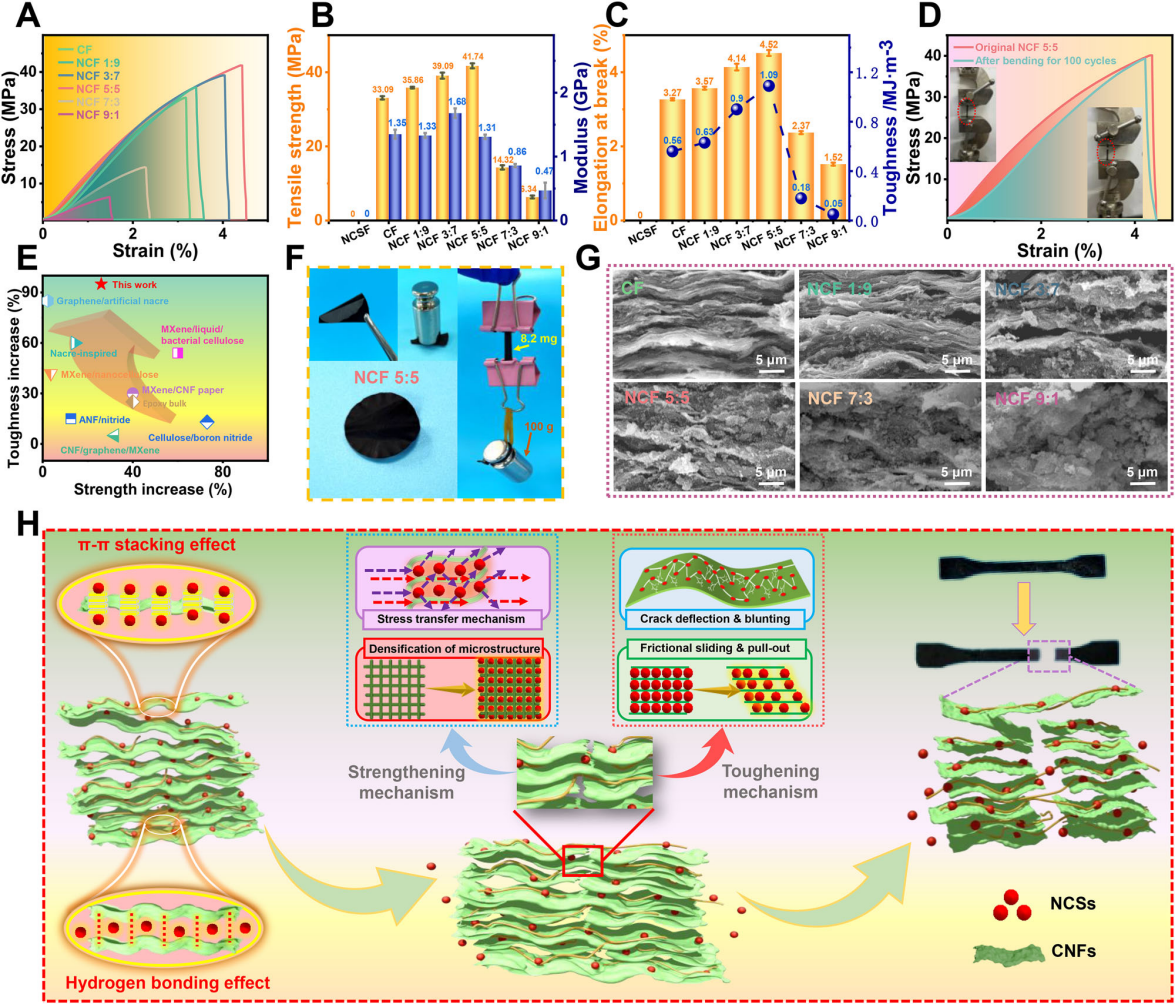
A) Stress–strain. B) Tensile strength and modulus. C) Elongation at break and toughness. D) Stress–strain of NCF 5:5 after 100 tensile. E) Comparison of strength and toughness enhancement rates with other reported composite films. F) Photographs of NCF 5:5 under mechanical deformation. G) SEM images of fracture surfaces. H) Strengthening and toughening mechanism.

The stress–strain curves presented in **Figure** **3**A illustrate the tensile behavior of CF, NCF, and NCSF under uniaxial tension. Both CF and NCF exhibit an initial linear elastic region, in which stress increases proportionally with strain during elastic deformation. After the curve reaches the maximum stress point, the stress drops sharply, corresponding to the fracture of the composite film. However, the stress–strain curve of NCSF could not be obtained due to the poor film‐forming ability of NCSs. Figure 3B presents the tensile properties of NCSF, CF, and NCF, including tensile strength and tensile modulus. The tensile strengths of CF, NCF 1:9, NCF 3:7, NCF 5:5, NCF 7:3, and NCF 9:1 are 33.09, 35.86, 39.09, 41.74, 14.32, and 6.34 MPa, respectively. The tensile modulus of CF, NCF 1:9, NCF 3:7, NCF 5:5, NCF 7:3, and NCF 9:1 is 1.35, 1.33, 1.68, 1.31, 0.86, and 0.47 GPa, respectively. It can be seen that both the tensile strength and tensile modulus first increase and then decrease, suggesting that the mechanical properties of the composite films are significantly influenced by the component ratio. As shown in Figure 3C, It can also be observed that the elongation at break of CF, NCF 1:9, NCF 3:7, NCF 5:5, NCF 7:3, and NCF 9:1 are 3.27%, 3.57%, 4.14%, 4.52%, 2.37%, and 1.52% and the toughness of CF, NCF 1:9, NCF 3:7, NCF 5:5, NCF 7:3, and NCF 9:1 are 0.56, 0.63, 0.90, 1.09, 0.18, and 0.05 MJ·m^−3^ showing that both the elongation at break and toughness initially increase with the addition of the NCSs component and then sharply decline beyond the optimal ratio. This trend suggests that a balanced composite formulation effectively enhances ductility and energy absorption due to improved interfacial bonding and stress distribution. However, the network structure may be destroyed due to stress concentration induced by the excessive addition of NCSs. NCF 5:5 exhibits the best tensile strength of 41.74 MPa, tensile modulus of 1.31 GPa, elongation at break of 4.52%, and toughness of 1.09 MJ·m^−3^, which can be attributed to the effective stress transfer and the continuous network structure constructed through the optimized component ratio of CNFs and NCSs in NCF. Furthermore, a 100‐cycle tensile test was conducted to evaluate the mechanical durability of NCF 5:5. As shown in Figure 3D, only slight changes are observed in the stress–strain curves, which indicate excellent cyclic stability and structural integrity of the composite film under repeated loading conditions. Figure 3E illustrates the percentage enhancement in tensile strength and toughness of the composite film compared to other composite films reported in previous studies. (MXene/liquid/bacterial cellulose,^[^ ^33^ ^]^ MXene/CNF paper,^[^ ^34^ ^]^ Epoxy bulk,^[^ ^35^ ^]^ ANF/nitride,^[^ ^36^ ^]^ CNF/graphene/MXene,^[^ ^37^ ^]^ MXene/nanocellulose,^[^ ^38^ ^]^ Nacre‐inspired,^[^ ^39^ ^]^ Graphene/artificial nacre^[^ ^40^ ^]^). As shown in Figure 3F, the prepared nanocomposite film can be folded into desired shapes and withstand external pressure without visible damage after unfolding. Remarkably, it is capable of lifting an object over 12195 times its own weight, demonstrating its exceptional mechanical robustness and flexibility. The cross‐sectional SEM images of CF and NCF in Figure 3G reveal distinct differences in microstructure. The CF exhibits a relatively loose and disordered layered structure with visible interlayer gaps, which may hinder effective stress transfer. With the gradual increase in NCSs content, the cross‐sectional structures of NCF 1:9 and NCF 3:7 become relatively denser compared to CF. However, interfacial delamination can still be observed, which indicates that a low NCSs content is insufficient to fully construct a continuous and robust network. In contrast, the NCF 5:5 shows a more compact and continuous cross‐sectional morphology, suggesting strong interfacial adhesion and an interconnected network structure that contributes to its superior mechanical performance. Conversely, the NCF 7:3 and NCF 9:1 display a fragmented and uneven morphology due to the fact that the network formation and structural integrity were disrupted by the excessive NCSs. These observations further support the optimal composition of NCF 5:5 for achieving balanced mechanical properties. Figure 3H illustrates the diverse reinforcement mechanisms contributing to the mechanical properties of NCF. First, the formation of a robust and integrated network structure was achieved by the *π–π* stacking interactions among NCSs and the hydrogen bonding among CNFs.^[^ ^41^ ^]^ Second, the tensile strength was enhanced by stress transfer mechanisms and densification of microstructure, and the toughening was achieved by crack deflection & blunting, and frictional sliding & pull‐out during the tensile process.^[^ ^42^ ^]^ Therefore, these synergistic mechanisms effectively contribute to the excellent mechanical properties of the NCF until structural failure occurs.

**Figure 4:**
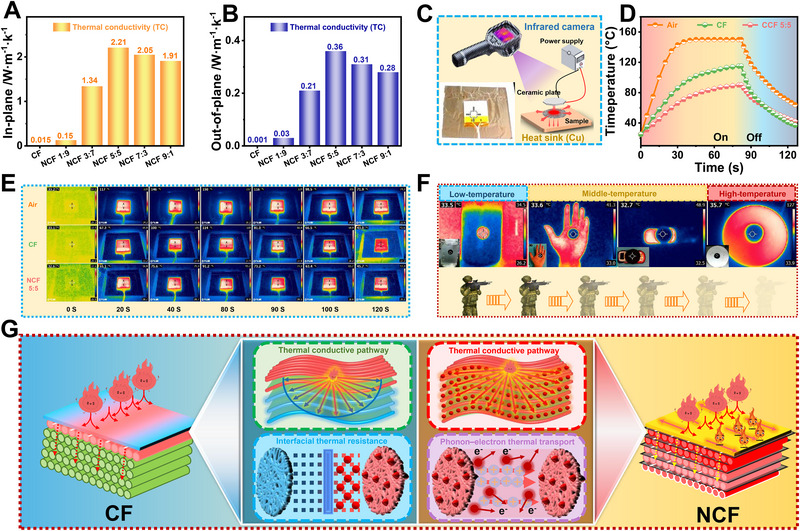
A) In‐plane thermal conductivity. B) Out‐of‐plane thermal conductivity. C) Setup demonstrating the tested sample as a heat spreader for a high‐power ceramic plate. D) Temperature rise curves. E) Infrared thermal images. F) Illustration of thermal camouflage. G) Thermal conduction mechanism.

**Figure** **4**A presents the in‐plane thermal conductivities of the NCF with different CNFs/NCSs ratios. It can be seen that the in‐plane TCs of CF, NCF 1:9, NCF 3:7, NCF 5:5, NCF 7:3, and NCF 9:1 are 0.015, 0.15, 1.34, 2.21, 2.05, and 1.91·W m^−1^·k^−1^. As the NCSs ratio increased from CF to NCF 5:5, the thermal conductivities improved from 0.015 to a peak of 2.21 W m^−1^·k^−1^, which can be attributed to the formation of a more continuous and interconnected thermally conductive network enabled by the synergistic effect of *π–π* stacking among NCSs and hydrogen bonding among CNFs. However, the thermal conductivities of NCF 7:3 and NCF 9:1 slightly decreased due to the fact that the uniform network was disrupted, and the interfacial thermal resistance increased due to excessive NCSs aggregation. As shown in Figure 4B, the out‐of‐plane thermal conductivities of CF, NCF 1:9, NCF 3:7, NCF 5:5, NCF 7:3, and NCF 9:1 are 0.001, 0.03, 0.21, 0.36, 0.31, and 0.28 W·m^−1^·K^−1^, respectively. This consistency confirms that the thermal transport behavior is governed by both the thermal conductivities of the fillers and the structural integration of the composite. Figure 4C shows a directional thermal conduction setup in which a ceramic heating plate is placed above the sample to provide uniform thermal radiation, while a copper heat sink (Cu) is positioned below to simulate heat dissipation. The effect of dissipating heat from the local heat source is demonstrated in Figure 4D,E, which presents the temperature–time curves under the heating setup for air, CF, and NCF 5:5. The surface temperature of the bare hot plate rises rapidly from 25 to 150°C within 80 s, then gradually decreases to 64°C over the next 40 s after the power is turned off. When CF is attached to the back of the hot plate, the temperature increases to 115°C in 80 s and drops to 37°C in 40 s. In contrast, with NCF 5:5, the surface temperature reaches only 90°C at 80 s and cools down to 42°C by 120 s. These results confirm the excellent potential of NCF 5:5 for high‐temperature thermal management in electronic devices. In addition, the thermal camouflage properties of NCF are evaluated at different environmental temperatures (Figure 4F). An NCF sample was attached to the sidewall of a beaker containing ice water for low low‐temperature test, and its measured radiation temperature was 33.5°C, which closely matched the ambient temperature. At moderate temperature, NCF samples applied to an experimenter's hand and a toy car both displayed surface temperatures similar to ambient conditions, effectively masking the underlying heat sources. Under high‐temperature conditions, an NCF sample was applied to a hot plate heated to 127°C, and the recorded temperature was only 35.7°C in the thermal image and showing a color similar to the ambient environment. In general, the NCF coverage area exhibits excellent thermal camouflage properties across a wide temperature range. Therefore, NCF is expected to minimize the infrared radiation disparity between military targets and their surroundings, thereby enhancing concealment. Figure 4G reveals the thermal conduction mechanism of the NCF formed through the synergistic integration of NCSs and CNFs. In the CF sample, thermal transfer was limited due to the lack of efficient thermal pathways and the presence of interfacial thermal resistance, resulting in poor thermal conductivity in both the in‐plane and through‐plane directions.^[^ ^43^ ^]^ In contrast, the NCF composite formed a more efficient and continuous thermal network through the synergistic integration of CNFs and NCSs, which facilitated enhanced heat transport.^[^ ^44^ ^]^ The phonon–electron thermal transport was enhanced and creating efficient thermal pathways in the in‐plane direction by NCSs. Therefore, the structural integration of CNFs and NCSs improved the overall thermal conductivity of the composite.^[^ ^45^ ^]^

**Figure 5:**
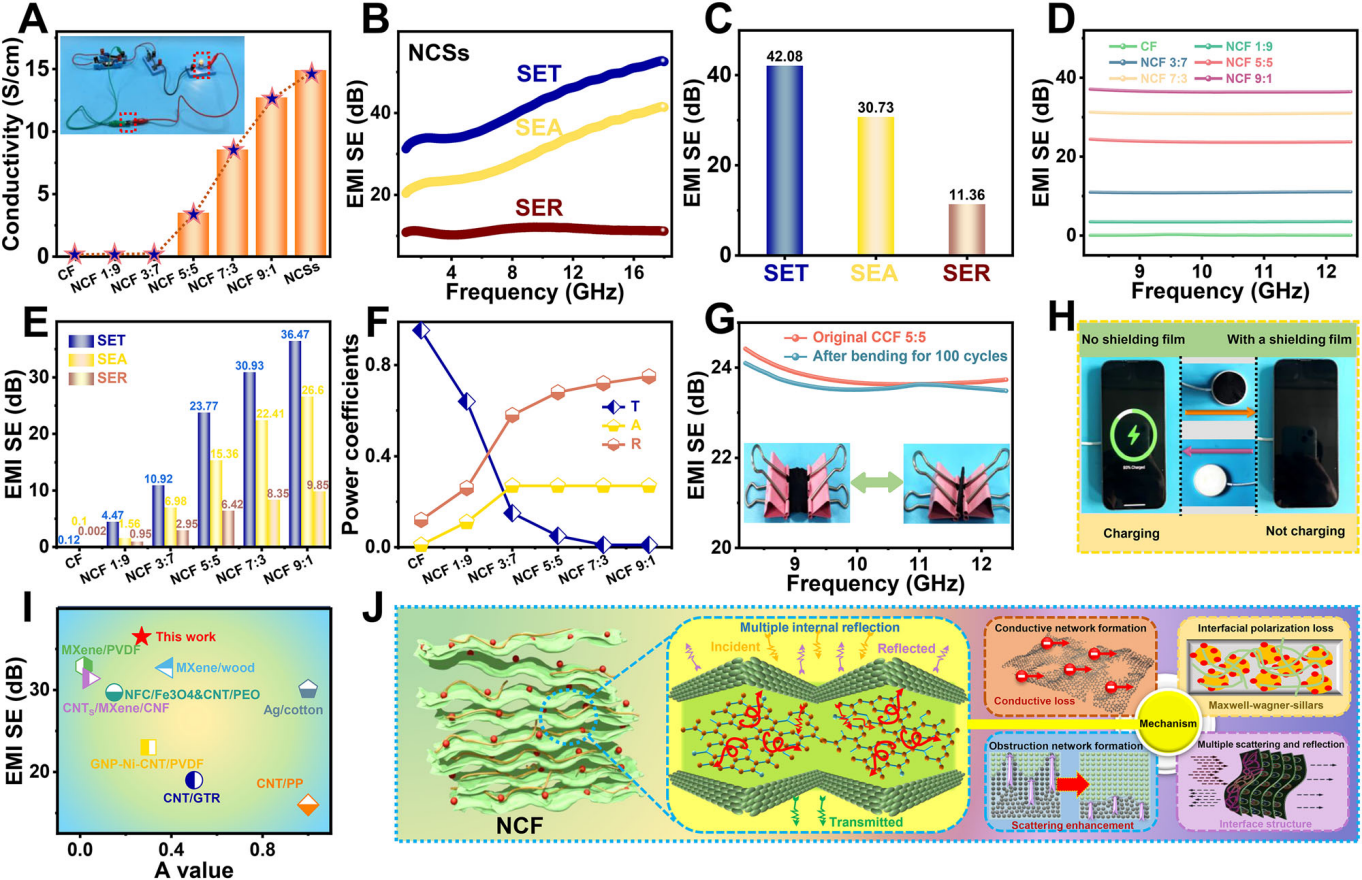
A) Electrical conductivity. B) EMI SE and C) SER, SEA, and SET. D) EMI SE and E) SER, SEA, and SET. F) Power coefficient (T: transmission, R: reflection, A: absorption). G) EMI SE of NCF 5:5 before and after 100 bending cycles. H) Practical demonstration of NCF shielding for wireless charging of a mobile phone. I) Comparison of EMI shielding effectiveness and the value of various composite films. J) EMI shielding mechanism.

**Figure** **5**A shows that the conductivity of NCF gradually increases with the increase of NCSs content. The electrical conductivity of NCF 5:5, NCF 7:3, NCF 9:1, and NCSs is 3.5, 8.56, 12.73, and 14.91 S cm^−1^, indicating the formation of a continuous and well‐connected conductive network in the NCF composites facilitated by the incorporation of NCSs. Figure 5B,C presents the total electromagnetic interference (EMI) shielding effectiveness (SET), shielding effect absorption (SEA), and shielding effect reflection (SER) for NCSs. It is observed that both the SET and SEA values increase steadily as the NCSs content increases, while SER remains relatively constant or exhibits only a slight increase in the whole X‐band (8.2–12.4 GHz), as shown in Figure 5B. The EMI shielding of the composite films originated from SER and SEA. SER referred to the electromagnetic waves reflected at the material surface due to impedance mismatch and free‐electron interactions, while SEA described the electromagnetic energy dissipated inside the material through conduction loss, dielectric loss, and multiple internal scattering. At low NCSs contents, the films exhibited limited electrical conductivity and fewer free electrons, leading to relatively weak conduction loss and reflection‐dominated shielding. As the NCSs content increased, a well‐developed conductive network was formed, which significantly enhanced electrical conductivity and conduction loss, thereby promoting electromagnetic wave absorption. Moreover, the increased interfacial polarization between NCSs and CNFs intensified dielectric loss. The hierarchical porous structure of the composites further induced multiple internal reflections and scattering, effectively extending the propagation path of electromagnetic waves and strengthening absorption. Consequently, with increasing NCSs concentration, the EMI shielding mechanism shifted from reflection‐dominated to absorption‐dominated, which aligned well with the observed shielding performance trends. The numerical values shown in Figure 5C indicate that the SET, SER, and SEA values for NCSs are 42.08, 11.36, and 30.73 dB, respectively, which suggests that the primary shielding mechanism for NCSs was absorption. As shown in Figure 5D, the EMI shielding properties of all composite films with different amounts of NCSs added are attributed to the formation of a more interconnected conductive network. Moreover, the SET shown in Figure 5E of CF, NCF 1:9, NCF 3:7, NCF 5:5, NCF 7:3, and NCF 9:1 is 0.12, 4.47, 10.92, 23.77, 30.93, and 36.47 dB, showing a clear trend of increasing shielding effectiveness with the rise in the NCSs content. It should be noted that SEA values generally increase for composites with higher NCSs content, suggesting that absorption plays a more significant role at higher NCSs concentrations. Meanwhile, the slow increase in SER values further explains that reflection still contributes to the overall shielding, though it plays a lesser role than SEA at higher NCSs content. Figure 5F depicts the relationship between the reflection (R), absorption (A), and transmission (T) power coefficients as the filler content varies. As can be seen that the T values of CF, NCF 1:9, and NCF 3:7 are relatively high, indicating that they do not possess electromagnetic shielding capabilities. In contrast, the T values of NCF 5:5, NCF 7:3, and NCF 9:1 are close to zero, which suggests that these materials can effectively block incident electromagnetic waves. It is worth noting that the R values are all greater than the A values in the NCF 5:5, NCF 7:3, and NCF 9:1, indicating that reflection contributes more than absorption to the overall EMI shielding in these NCF. The EMI shielding properties of the composite film after 100 cycles of repeated bending, shown in Figure 5G, present negligible variation compared to the initial state, indicating excellent mechanical flexibility and structural stability of the composite. The NCF 9:1 is applied to a wireless charging device to verify its EMI shielding under practical conditions, as shown in Figure 5H. The suppression of charging indicates effective attenuation of near‐field electromagnetic interference due to enhanced energy dissipation via conduction, dielectric loss, and multiple internal reflections in the conductive network and porous structure of the composite. These mechanisms disrupt electromagnetic coupling, which confirms the practical shielding potential of the film in portable and wearable electronics. It was found that the smartphone started charging immediately (indicated by the green circles and charging icon) upon being placed on the wireless charger. However, the transmission of radio waves was effectively blocked after introducing the NCF 9:1, causing the charging process to stop. This demonstrates that the composite film can efficiently shield external electromagnetic radiation. As shown in Figure 5I, the NCF 9:1 exhibits an outstanding EMI shielding effectiveness of 36.47 dB, which not only surpasses the commercial requirement of 20 dB but also outperforms many previously reported biomass‐based or carbon nanomaterial‐based composite films (MXene/PVDF, CNTs/MXene/CNF, NFC/Fe3O4&CNT/PEO, MXene/wood, Ag/cotton, CNT/GTR, CNT/PP, GNP‐Ni‐CNT/PVDF).^[^ ^46^ ^]^ Figure 5J demonstrates the excellent EMI shielding properties of the NCF, which stem from multiple synergistic mechanisms involving both the composition and structural features of the material. NCSs with high electrical conductivity and partially graphitized domains play a dominant role in forming a continuous and efficient conductive network throughout the matrix of CNFs.^[^ ^47^ ^]^ The conductive network formed by the NCF efficiently reflects incident electromagnetic waves at the surface and dissipates their energy through conductive loss and multiple internal reflections. The graphitic carbon domains within the NCSs structure further contribute to the attenuation of electromagnetic energy by promoting conductive loss via an extended π‐conjugated system. In parallel, the porous and entangled structure of the CNFs matrix introduces multiple scattering centers and interfaces, which increase the propagation path and thereby enhance the probability of electromagnetic wave attenuation. Moreover, the mechanical entanglement of fibers contributes to forming a tortuous network that physically impedes the direct transmission of electromagnetic waves.^[^ ^48^ ^]^ Furthermore, the abundant heterogeneous interfaces between conductive NCSs and insulating CNFs lead to significant interfacial polarization under alternating fields. This dielectric relaxation, coupled with the Maxwell–Wagner–Sillars (MWS) effect, causes charge accumulation at phase boundaries, which enhances dipolar polarization loss and contributes to the absorption component of the shielding effectiveness.^[^ ^49^ ^]^ The hierarchical porous structure not only facilitates multiple internal reflections but also amplifies these polarization effects by increasing the interface density. Overall, the high EMI shielding effectiveness of NCF is a result of the synergistic integration of conductive loss, dielectric loss, and physical scattering, demonstrating the critical role of both composition and structural design in tuning EMI property.

## 4 Conclusion

All‐biomass nanocomposite films were fabricated utilizing a facile vacuum‐assisted filtration strategy through the synergistic integration of NCSs and CNFs. The study systematically investigated the structural evolution and interfacial interactions that underpin the enhanced properties of the NCF composites. It was found that the facilitated efficient charge transport pathways and mechanical reinforcement were attributed to the interpenetrating architecture and *π–π* stacking between NCSs and CNFs, thereby endowing the composite films with superior thermal conductivity property and EMI shielding. The prepared NCF 5:5 exhibits a tensile strength of 41.74 MPa, a tensile modulus of 1.31 GPa, an elongation at break of 4.52%, a tensile toughness of 1.09 MJ·m^−3^, an in‐plane thermal conductivity of 2.21 W·m^−1^·K^−1^, and an EMI shielding of 23.77 dB. In addition, it is interesting to note that the remarkable thermal camouflage capability and the maintained excellent mechanical and EMI shielding stability after 100 cycles were achieved. Therefore, the synergistic effects of hierarchical structuring and molecular‐level interactions not only enhanced the functional performance of the films but also demonstrated the potential of biomass‐derived materials in high‐performance, sustainable electronic applications. Possessing sustainability, low cost, and multifunctionality of biomass‐based nanocomposite films in this study provide a promising and potential strategy in next‐generation flexible electronics and environmentally friendly EMI shielding solutions.

## Conflict of Interest

The authors declare no conflict of interest.

## Supporting information



Supporting Information

## Data Availability

Research data are not shared.
