# Supplementary material for: All‐Biomass Nanocomposite Films via Facile and Sustainable Design Procedure for Thermal Management and Electromagnetic Interference Shielding
Source: Adv Sci (Weinh). 2025 Aug 27;12(43):e10372. doi: 10.1002/advs.202510372 (PMC12631880; doi:10.1002/advs.202510372)
Supplement: Supplementary file 1 — Supporting Information [file ADVS-12-e10372-s001.docx]

**Supporting Information**

**All-biomass nanocomposite films via facile and sustainable design procedure for thermal management and** **electromagnetic interference shielding**

Junchao Ren^a^, Chenglei Huang^a^, Rui Tan^a^, Jianlong Chen^a^, Mengde Huang^a^, Mingfeng Wang^a^, Weiwei Liu^a^, Bin Li^a^, Zhong Ma^a^, Lu Wang^b^, Hanwu Lei^c^, Erguang Huo^d^, Qingfa Zhang^a^*

*^a^School of Engineering, Anhui Agricultural University, Hefei, Anhui 230036, China*

*^b^School of Food and Biological Engineering, Hefei University of Technology, Hefei, Anhui 230009, China*

*^c^Department of Biological Systems Engineering, Washington State University, Richland, Washington 99354, United States*

*^d^Key Laboratory of Efficient Low-carbon Energy Conversion and Utilization of Jiangsu Provincial Higher Education Institutions, School of Physical Science and Technology, Suzhou University of Science and Technology, Suzhou, Jiangsu 215009, China*

∗Corresponding author: *Email address: zhangqingfa@ahau.edu.cn (Q. Zhang)*

**Table of Contents**

**Supplementary Figures and Tables**

Fig. S1. The main idea of this study

Fig. S2. (A) Photograph of the self-developed NHTPR. (B) Photograph of the furnace during operation. (C) Photograph of the interior of the furnace chamber. (D) Schematic diagram of the NHTPR

Fig. S3. SEM images of the surface morphology of the composite films

Fig. S4. SEM images of the tensile fracture surfaces of CF and NCF5:5

Fig. S5. AFM height images

Fig. S6. (A) Unit cell model. (B) Blended box model of NCF. (C) Temperature convergence curve of

the simulation box. (D) Energy convergence curve of the simulation box. (E) Parameter convergence curves. (F) Total energy convergence curve

Fig. S7. (A) Unit cell model. (B) Blended box model of NCF. (C) Temperature convergence curve of

the simulation box. (D) Energy convergence curve of the simulation box. (E) Parameter convergence curves. (F) Total energy convergence curve

Fig. S8. (A) Graphene structure and transmission spectrum. (B) Graphene-like structure and smoother

transmission spectrum

Fig. S9. Photographs of films after 0, 5, 10, and 15 days under ambient conditions

Fig. S10. Photographs of films after 0 h, 3 h, 6 h, and 12 h under ambient conditions

Fig. S11. Photographs of films after 24 h at different temperatures (10°C, 30°C, 50°C, 70°C)

Fig. S12. Photographs of bacterial growth inhibition tests with the films

Fig. S13. DSC curves

Fig. S14. Plots of EMI shielding effectiveness (SET), reflection (SER), and absorption (SEA) curves

with corresponding values under different conditions: (A) Repeated bending up to 500 cycles. (B) Thermal aging at 60°C for 24 h. (C) UV exposure for 24 h. (D) Ambient storage for 30 days. (E) Summary table comparing the original sample and the above four conditions

Table S1. Comparison of different carbon material preparation methods in terms of temperature, batch

capacity, energy consumption, and cost

Table S2. Multi-parameter comparison of composite films and commercial EMI shielding

Table S3. Comparison of unit costs for various carbon materials used in EMI shielding applications

Table S4. Cost comparison of cellulose-based EMI shielding films using different carbon materials

Table S5. Comparative analysis of composite film performance with existing EMI shielding materials

Table S6. Average thickness and EMI shielding property of CF and NCF


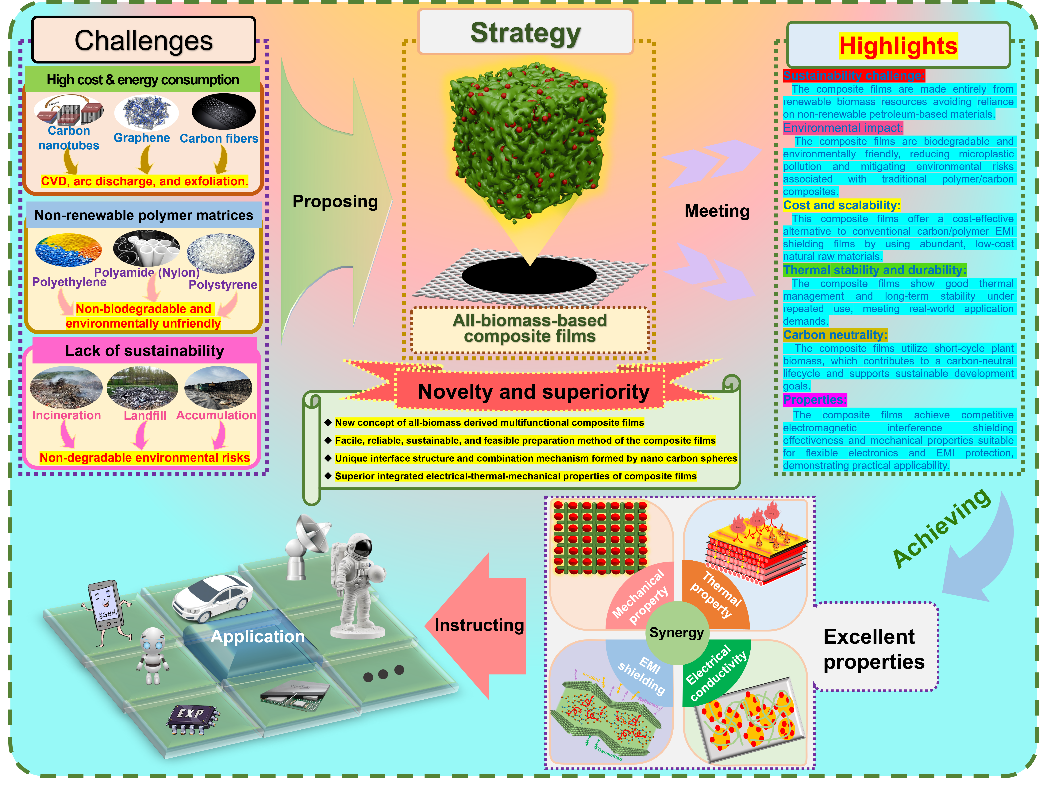


Fig. S1. The main idea of this study


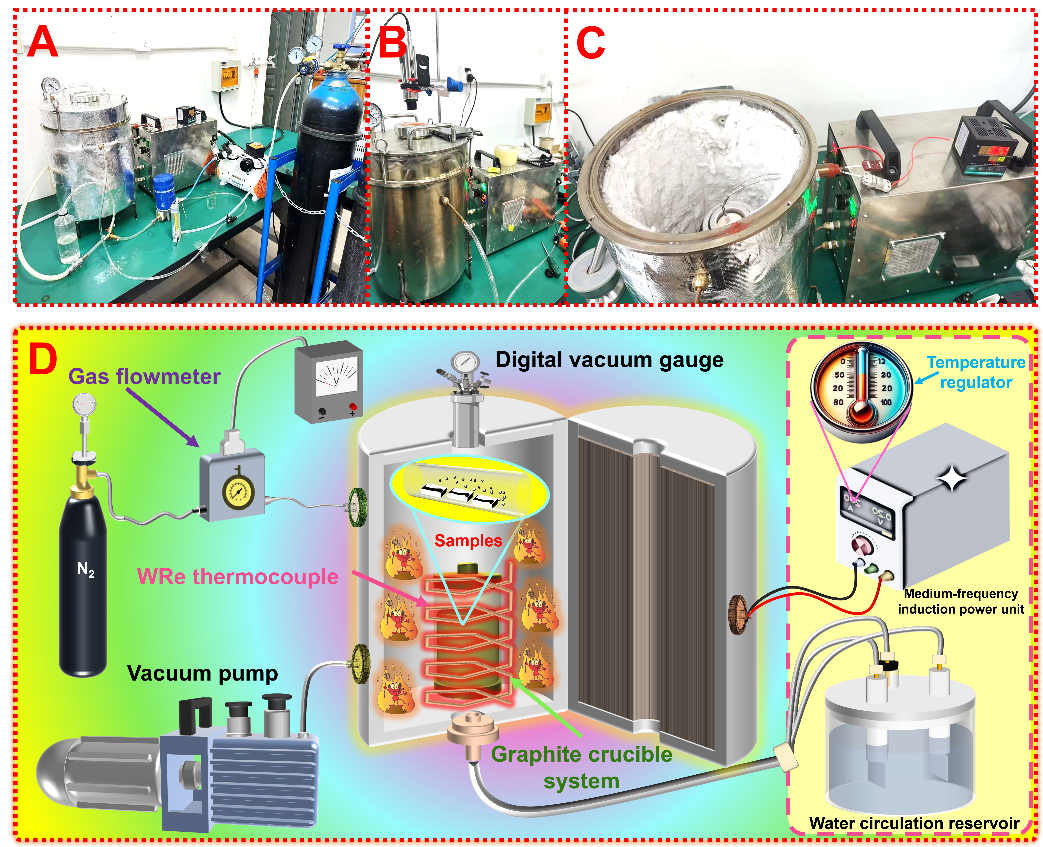


Fig. S2. (A) Photograph of the self-developed NHTPR. (B) Photograph of the furnace during operation. (C) Photograph of the interior of the furnace chamber. (D) Schematic diagram of the NHTPR


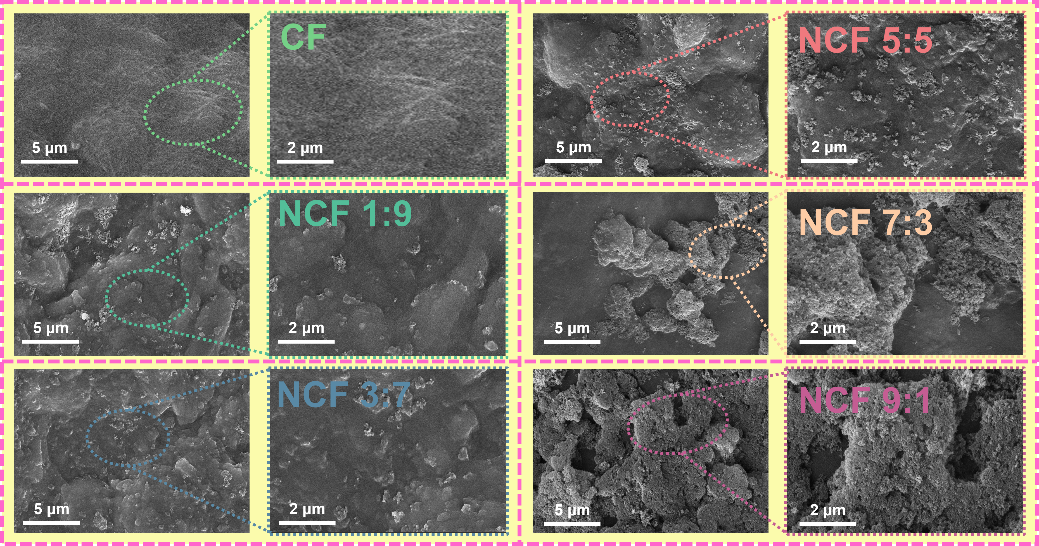


Fig. S3. SEM images of the surface morphology of the composite films

Fig. S4. SEM images of the tensile fracture surfaces of CF and NCF5:5


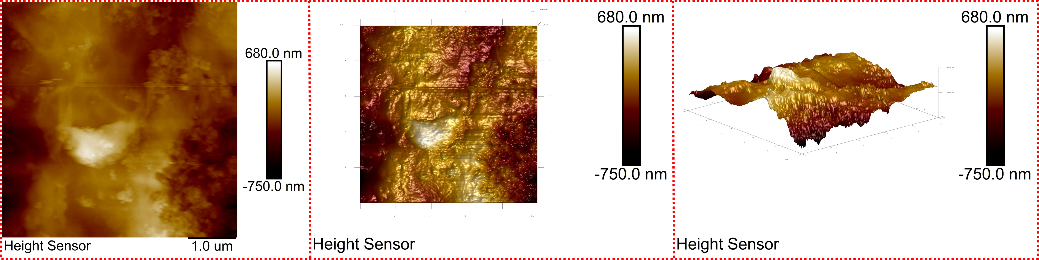


Fig. S5. AFM height images


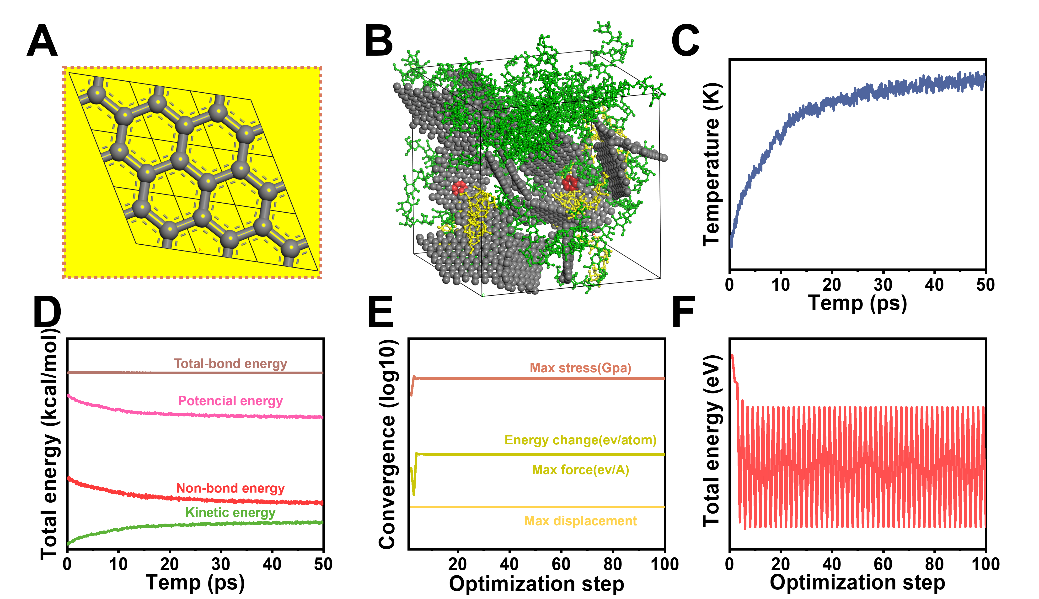


Fig. S6. (A) Unit cell model. (B) Blended box model of NCF. (C) Temperature convergence curve of the simulation box. (D) Energy convergence curve of the simulation box. (E) Parameter convergence curves. (F) Total energy convergence curve


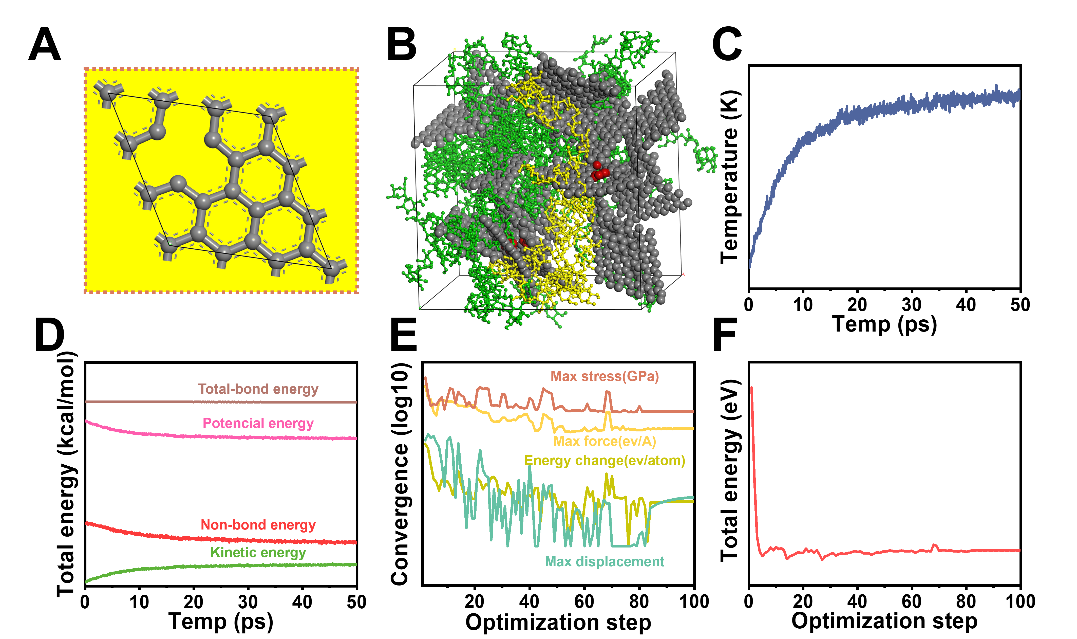


Fig. S7. (A) Unit cell model. (B) Blended box model of NCF. (C) Temperature convergence curve of the simulation box. (D) Energy convergence curve of the simulation box. (E) Parameter convergence curves. (F) Total energy convergence curve


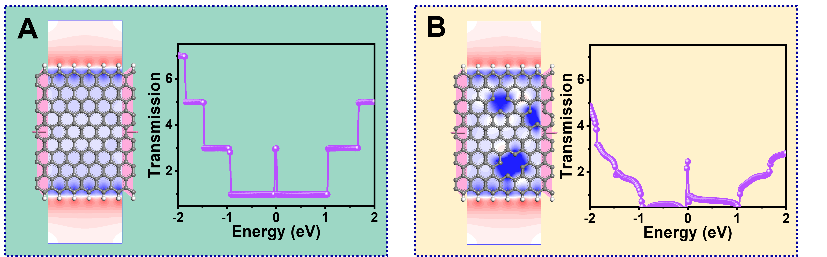


Fig. S8. (A) Graphene structure and transmission spectrum. (B) Graphene-like structure and smoother transmission spectrum

**
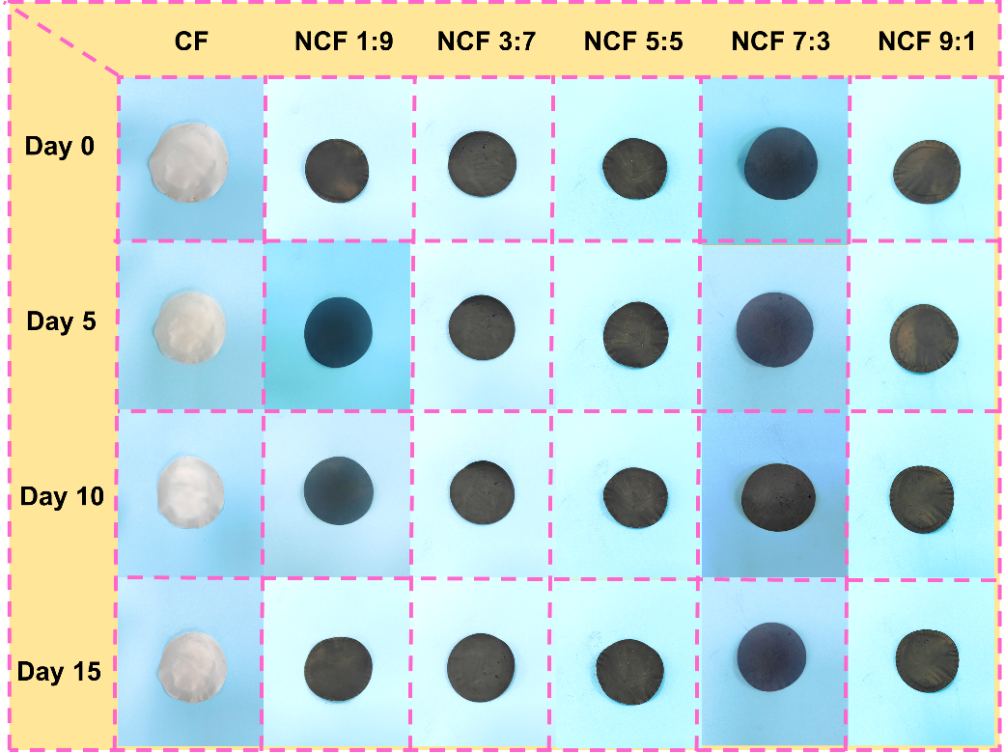
**

Fig. S9. Photographs of films after 0, 5, 10, and 15 days under ambient conditions


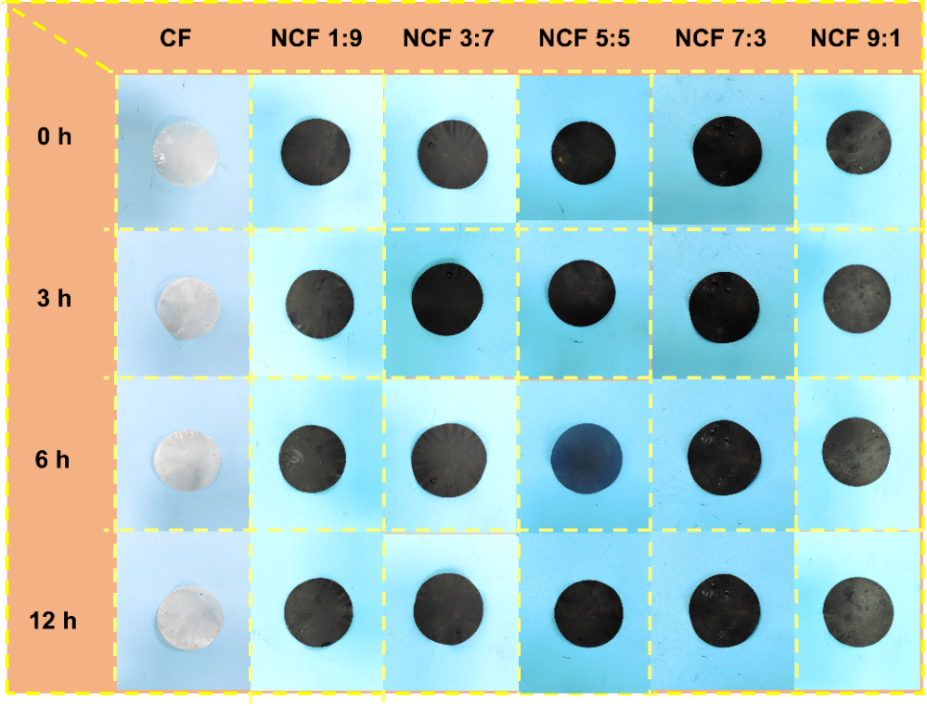


Fig. S10. Photographs of films after 0 h, 3 h, 6 h, and 12 h under ambient conditions


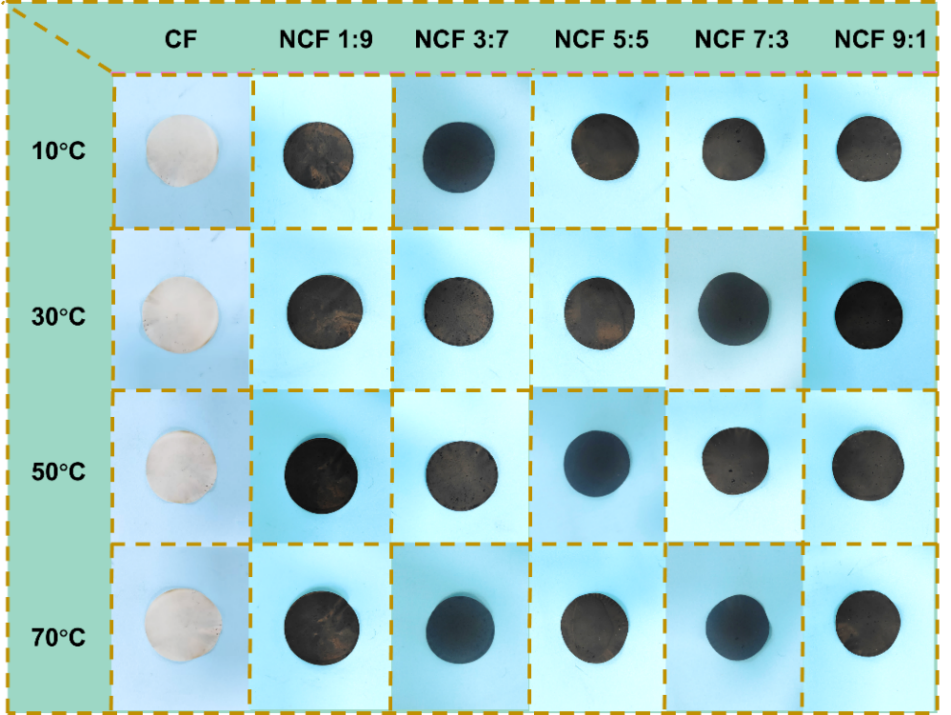


Fig. S11. Photographs of films after 24 h at different temperatures (10°C, 30°C, 50°C, 70°C)

Fig. S12. Photographs of bacterial growth inhibition tests with the films

Fig. S13. DSC curves


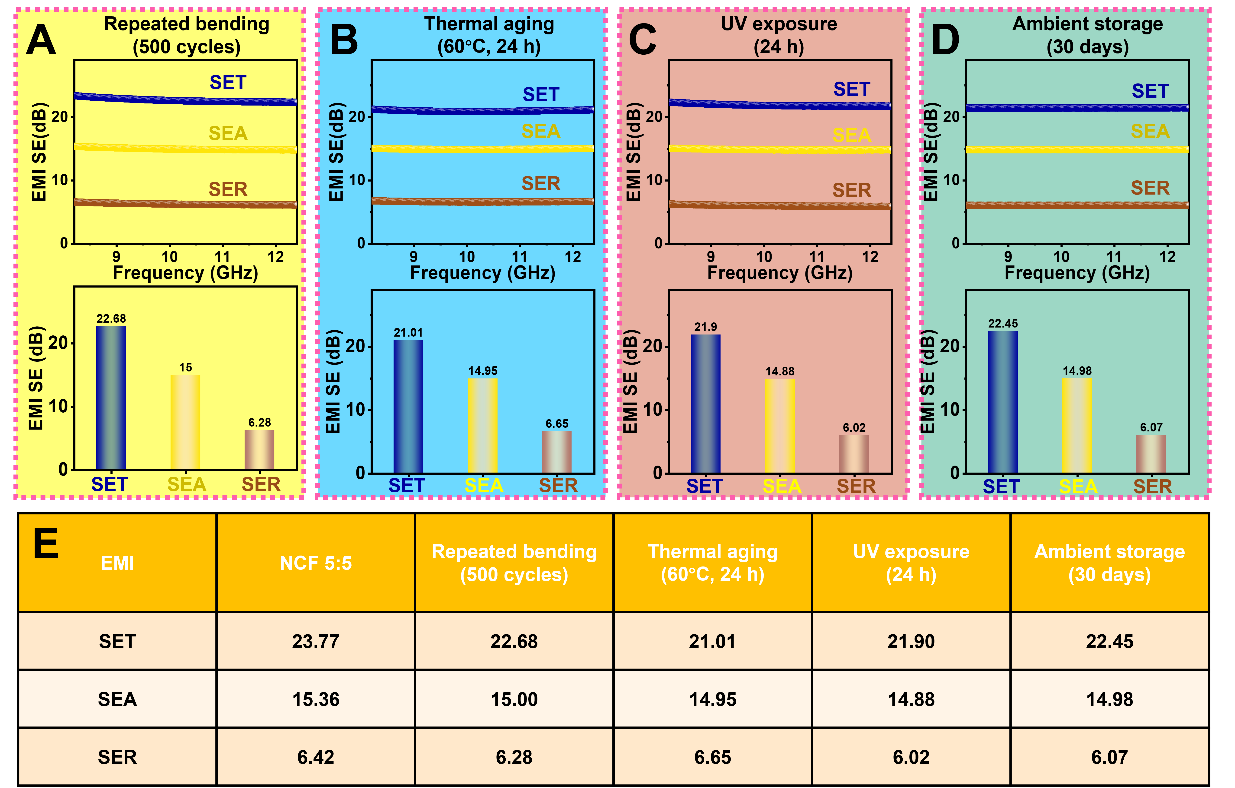


Fig. S14. Plots of EMI shielding effectiveness (SET), reflection (SER), and absorption (SEA) curves with corresponding values under different conditions: (A) Repeated bending up to 500 cycles. (B) Thermal aging at 60°C for 24 h. (C) UV exposure for 24 h. (D) Ambient storage for 30 days. (E) Summary table comparing the original sample and the above four conditions

**Table S1.** Comparison of different carbon material preparation methods in terms of temperature, batch capacity, energy consumption, and cost

| Methods/Equipments | Maximum carbonization temperature (°C) | Maximum batch size (g) | Rated power (kW) | Energy per batch (kWh) | Energy per gram (kWh/g) | Remarks |
| --- | --- | --- | --- | --- | --- | --- |
| Commercial vacuum tube furnace  (e.g., LabX T1200) | 1200 | 1-5 | ~5 | ~10 | 1.0-2.0 | Limited capacity and temperature  Higher unit energy consumption |
| Arc discharge method | 3500 | ~0.5 | 20-25 | ~40 | 60-70 | Extremely low throughput  High power usage |
| Liquid-phase exfoliation | 60 | 0.5-5 | 1-2 | 5-10 | ~2 | Solvent-heavy  Low yield  Poor scalability |
| Chemical vapor deposition (CVD) | 1000 | 1-3 | 10-20 | ~30 | 15-35 | Costly and resource-intensive  Time-consuming and sluggish  Hard to expand or scale |
| Self-developed NHTPR | **2200** | **~35** | **~5.5** | **~10** | **~0.29** | **High temperature operation Large batch size processing Scalable design**  **Energy-efficient performance** |

**Table S2.** Multi-parameter comparison of composite films and commercial EMI shielding

| Materials | EMI shielding (dB) | Cost | Processability | Environmental friendliness | Density | Main drawbacks | References |
| --- | --- | --- | --- | --- | --- | --- | --- |
| Metals (Cu, Al) | 50–80 | High | Good | Poor | High | Heavy, corrosive, rigid | [1] |
| Carbon nanotubes  /graphene | 30–70 | Very high | Moderate | Moderate | Low | Expensive, complex synthesis | [2] |
| Magnetic Materials (e.g., Ferrites) | 20–40 | Moderate | Fossil-derived | Moderate | Medium–High | Brittle, low-frequency, limited | [3] |
| Conductive Fabrics (e.g., metal-coated textiles) | 30–60 | Medium–high | Excellent | Poor | Low | Still costly, mechanical durability issues | [4] |
| Traditional Carbon Materials (Carbon black, Graphite) | 15–30 | Low | Moderate | Acceptable | Medium | Energy-intensive, inconsistent quality | [5] |
| Previous biomass-derived carbon materials | 20–50 | Low | Poor | Moderate | Low | Low shielding performance | [6] |
| NCF | **30-45** | **Low** | **Easily processable, formable** | **Green & sustainable** | **Lightweight** | **/** | **This work** |

**Table S3.** Comparison of unit costs for various carbon materials used in EMI shielding applications

| Materials | Market price range (USD/g) | Remarks | Sources/References |
| --- | --- | --- | --- |
| Graphene (CVD method) | 41.7 – 138.9 | High-purity; lab-grade; extremely expensive | Xiamen Kennah Nano Materials Co., Ltd. |
| Graphene powder (liquid exfoliation) | 13.9 – 41.7 | Industrial grade; available online | Ningbo Moxi Technology Co., Ltd. |
| Multi-walled carbon nanotubes | 27.8 – 111.1 | Multi-walled carbon nanotubes | Xiamen Refine Nano Materials Co., Ltd. |
| Single-walled carbon nanotubes | 69.4 – 277.8 | Single-walled; high purity; very costly | Guangzhou Zhongke Nano Technology Co., Ltd. |
| Conductive carbon black | 0.14 – 0.42 | Low cost; limited EMI performance | Anhui Zhongke Carbon Black Co., Ltd. |
| Industrial graphite powder | 7.30 – 10.50 | Economical but poor conductivity | Jiaozuo Jinniu Graphite Co., Ltd. |
| PAN-based carbon fibers | 0.69 – 2.78 | Mainly used for structural reinforcement | Zhongfu Shenying Carbon Fiber Co., Ltd. |
| Carbon materials (reported previously) | 0.14 – 1.39 | Low-cost, but low yield and higher energy usage | [7] |
| This work | **~ 0.06** | **Laboratory-fabricated using agricultural waste; low energy and cost.** | **Laboratory-synthesized** |

**Table S4.** Cost comparison of cellulose-based EMI shielding films using different carbon materials

| Commercial EMI shielding materials | | Price range (USD/m²) | Main suppliers/Channels | | | Remarks |
| --- | --- | --- | --- | --- | --- | --- |
| Metal foil shielding films | | 10 – 50 | 3M company | | | Copper foil, aluminum foil; good shielding but heavier |
| Carbon nanotube composite films | | 20 – 100 | Alibaba group | | | Lightweight, flexible, high performance, relatively expensive |
| Graphene-based films | | 50 – 200 | Graphenea S.A. | | | High-end materials for demanding applications |
| Conductive polymer films (PEDOT:PSS) | | 30 – 120 | Sigma-Aldrich corporation | | | Flexible, transparent, medium price |
| Carbon black composite films | | 5 – 30 | Alibaba group | | | Low cost, suitable for large-area use |
| Composite EMI shielding  materials | **Estimated cost (USD/g)** | | | **Carbon material source/equipment used** | **Main supplier/Reference source** | **Remarks** |
| CNT/CNF | 0.20 – 0.50 | | | Commercial MWCNTs (CVD or catalytic method) | Timesnano (Chengdu Organic Chemicals Co., Ltd.) | High cost, excellent conductivity, limited scalability |
| SWCNT/CNF  Graphene | 0.50 – 1.00 | | | High-purity SWCNTs from commercial suppliers | Meijo Nano Carbon Co., Ltd. | High shielding, but expensive and hard to disperse |
| Graphene/CNF | 0.30 – 0.80 | | | CVD-grown graphene | Graphenea S.A. | Good performance, high purity, low scalability |
| Graphene/CNF | 0.10 – 0.30 | | | Liquid-exfoliated graphene | ACS Material, LLC | Lower cost than CVD, suitable for bulk processing |
| Carbon black/CNF | 0.15 – 0.35 | | | Commercial conductive carbon black | Cabot Corporation | Low-cost filler, modest EMI shielding |
| This work | **~0.03** | | | **Biomass-based NCSs, self-built furnace** | **Self-prepared** | **Environmentally friendly, low cost** |

**Table S5.** Comparative analysis of composite film performance with existing EMI shielding materials

| Studies | Filler type | EMI shielding (dB) | Tensile Strength (MPa) | Conductivity (S/m) | Shielding Mechanism | Process complexity | Biodegradability | Cost | References |
| --- | --- | --- | --- | --- | --- | --- | --- | --- | --- |
| Carbon nanotubes (CNTs)/ carbon fibers | CNTs | 30-40 | ~25 | / | Reflection-dominated | High (Custom CVD design) | Low | High | [8] |
| CCA@rGO/polydimethylsiloxane (PDMS) | rGO | 13-30 | / | ~400 | Mixed | High (vacuum impregnation, freeze-drying, thermal annealing) | Low | High | [9] |
| MXene frameworks | MXene | 25-50 | ~20 | ~800 | Reflection-dominated | Very High (Chemically cross-linked) | Low | High | [10] |
| Double-walled carbon nanotube film (DWCNTF) | Carbon nanotube | 50-70 | ~37 | / | Absorption-dominated | High (Electromagnetic wave propagation theorie) | Low | High | [11] |
| AgNW@rGO networks | rGO + Ag nanowires | 30-45 | / | ~1500 | Reflection-dominated | High (Pulsed laser irradiation treatment) | Low | High | [12] |
| WPC composites | Biochar | 10-25 | / | ~160 | Absorption-dominated | ~0.34 | High | High | [13] |
| NCF | **Nano carbon spheres** | **25-40** | **~42** | **~1200** | **Absorption-dominated** | **Low (Vacuum-assisted self-assembly)** | **High** | **Low** | **This work** |

**Table S6.** Average thickness and EMI shielding properties of CF and NCF

| Samples | CF | NCF 1:9 | NCF 3:7 | NCF 5:5 | NCF7:3 | NCF 9:1 |
| --- | --- | --- | --- | --- | --- | --- |
| Average  thickness (mm) | 0.18±0.02 | 0.20±0.01 | 0.19±0.01 | 0.22±0.02 | 0.25±0.04 | 0.26±0.03 |
| EMI shielding (dB) | 0.12 | 4.47 | 10.92 | 23.77 | 30.93 | 36.47 |

**Additional References**

1. Y. Feng, G. Polizos, S. Kalnaus, R. Tao, S. Neumayer, W. Steenman, J. Sharma, D. J. Pereira, B. Morin, J. Li, Energy Environ. Mater. 2025, 8, e12878.
2. W. Feng, L. Zou, C. Lan, S. E, X. Pu, Adv. Fiber Mater. 2024, 6, 1657.
3. F. Hu, M. Kui, J. Zeng, P. Li, T. Wang, J. Li, B. Wang, C. Wu, K. Chen, ACS Nano 2024, 18, 25852.
4. W. Tao, W. Shao, M. Ma, S. Chen, Y. Shi, H. He, Y. Zhu, X. Wang, Nano Mater. Sci. 2025, 7, 65.
5. Y. Deng, Y. Chen, W. Liu, L. Wu, Z. Wang, D. Xiao, D. Meng, X. Jiang, J. Liu, Z. Zeng, others, Carbon Energy 2024, 6, e593.
6. Y. Yue, D. Zhang, P. Wang, X. Xia, X. Wu, Y. Zhang, J. Mei, S. Li, M. Li, Y. Wang, others, Adv. Mater. 2024, 36, 2313971.
7. M. Islam, C. Selhuber-Unkel, J. G. Korvink, A. D. Lantada, Matter 2023, 6, 1382.
8. Q. Men, S. Wang, Z. Yan, B. Zhao, L. Guan, G. Chen, X. Guo, R. Zhang, R. Che, Adv. Compos. Hybrid Mater. 2022, 5, 2429.
9. P. Song, B. Liu, C. Liang, K. Ruan, H. Qiu, Z. Ma, Y. Guo, J. Gu, Nano-Micro Lett. 2021, 13, 91.
10. S. Lee, T. Y. Ko, J. Hong, A. S. Lee, J.-S. Lee, S. J. Kim, Compos. Part B Eng. 2024, 287, 111847.
11. B. Park, S. Hwang, H. Lee, Y. Jung, T. Kim, S. J. Kwon, D. Jung, S. Lee, Adv. Funct. Mater. 2024, 34, 2406197.
12. Y. Yang, S. Chen, W. Li, P. Li, J. Ma, B. Li, X. Zhao, Z. Ju, H. Chang, L. Xiao, others, Acs Nano 2020, 14, 8754.
13. C. Liang, H. Qiu, P. Song, X. Shi, J. Kong, J. Gu, Sci. Bull. 2020, 65, 616.
